# KIR2DS5 allotypes that recognize the C2 epitope of HLA‐C are common among Africans and absent from Europeans

**DOI:** 10.1002/iid3.178

**Published:** 2017-07-06

**Authors:** Jeroen H. Blokhuis, Hugo G. Hilton, Lisbeth A. Guethlein, Paul J. Norman, Neda Nemat‐Gorgani, Annettee Nakimuli, Olympe Chazara, Ashley Moffett, Peter Parham

**Affiliations:** ^1^ Departments of Structural Biology and Microbiology and Immunology Stanford University Stanford CA USA; ^2^ Department of Obstetrics and Gynaecology Makerere University Kampala Uganda; ^3^ Department of Pathology and Centre for Trophoblast Research University of Cambridge Cambridge United Kingdom

**Keywords:** Africa, HLA‐C, KIR, NK cells, pregnancy

## Abstract

**Introduction:**

KIR2DS5 is an activating human NK cell receptor of lineage III KIR. These include both inhibitory KIR2DL1, 2 and 3 and activating KIR2DS1 that recognize either the C1 or C2 epitope of HLA‐C. In Europeans KIR2DS5 is essentially monomorphic, with KIR2DS5*002 being predominant. Pioneering investigations showed that KIR2DS5*002 has activating potential, but cannot recognize HLA‐A, ‐B, or ‐C. Subsequent studies have shown that KIR2DS5 is highly polymorphic in Africans, and that KIR2DS5*006 protects pregnant Ugandan women from preeclampsia. Because inhibitory C2‐specific KIR2DL1 correlates with preeclampsia, whereas activating C2‐specific KIR2DS1 protects, this association pointed to KIR2DS5*006 being an activating C2‐specific receptor. To test this hypothesis we made KIR‐Fc fusion proteins from all ten KIR2DS5 allotypes and tested their binding to a representative set of HLA‐A, ‐B and ‐C allotypes.

**Results:**

Six African‐specific KIR2DS5 bound to C2**^+^**HLA‐C but not to other HLA class I. Their avidity for C2 is ∼20% that of C2‐specific KIR2DL1 and ∼40% that of C2‐specific KIR2DS1. Among the African C2 receptors is KIR2DS5*006, which protected a cohort of pregnant Ugandans from pre‐eclampsia. Three African KIR2DS5 allotypes and KIR2DS5*002, bound no HLA‐A, ‐B or ‐C. As a group the C2‐binding KIR2DS5 allotypes protect against pre‐eclampsia compared to the non‐binding KIR2DS5 allotypes. Natural substitutions that contribute to loss or reduction of C2 receptor function are at positions 127, 158, and 176 in the D2 domain.

**Conclusions:**

KIR2DS5*005 has the *KIR2DS5* consensus sequence, is the only allele found at both centromeric and telomeric locations of *KIR2DS5*, and is likely the common ancestor of all *KIR2DS5* alleles. That KIR2DS5*005 has C2 receptor activity, points to KIR2DS5*002, and other allotypes lacking C2 receptor function, being products of attenuation, a characteristic feature of most *KIR B* haplotype genes. Alleles encoding attenuated and active *KIR2DS5* are present in both centromeric and telomeric locations.

## Introduction

Killer cell immunoglobulin‐like receptors (KIR) are expressed by subsets of human NK cells, a subpopulation of lymphocytes that contributes to innate immunity, adaptive immunity and reproduction 1, 2. The target cell ligands recognized by KIR are epitopes of HLA‐A, ‐B and ‐C. These epitopes are defined by alternative sequence motifs at residues 76–83 of the α_1_ domain. The human KIR family comprises four phylogenetic lineages, of which lineage III includes all KIR that recognize the C1 and C2 epitopes of HLA‐C. These epitopes are defined by dimorphism at position 80 of HLA‐C, where asparagine confers C1 specificity and lysine confers C2 specificity 3.

KIR2DL1 is an inhibitory receptor that is highly specific for C2 whereas KIR2DL2 and KIR2DL3 are inhibitory receptors that principally recognize C1 4. Moreover, KIR2DL2, and to lesser extent KIR2DL3, cross‐react with some C2^+^HLA‐C 5, 6. In addition to these much‐studied inhibitory lineage III receptors, there are five activating lineage III receptors: KIR2DS1, 2DS2, 2DS3, 2DS4, and 2DS5. In comparison to the inhibitory receptors, these activating receptors are not as well characterized 7, 8. Only KIR2DS1 has been shown to recognize HLA‐C with specificity like that of an inhibitory KIR. Like inhibitory KIR2DL1, KIR2DS1 has methionine 44 and is specific for the C2 epitope. But the avidity of KIR2DS1 for C2 is about half that of KIR2DL1 7, 8.

Epidemiological studies have correlated the presence or absence of the *KIR2DS1* gene with several pregnancy syndromes 9, 10, 11. In these and other disease association studies, it has proved useful to divide the numerous *KIR* haplotypes into two groups, *KIR A* and *KIR B*, based upon their content of activating *KIR* genes. *KIR A* haplotypes can have either no activating *KIR* genes, a functional form of the *KIR2DS4* gene or a non‐functional form of the *KIR2DS4* gene. In contrast, *KIR B* haplotypes can have many different combinations of the five *KIR2DS*, as well as *KIR3DS1*. Pregnant women who have a *KIR A*/*A* genotype are at greater risk for pre‐eclampsia than pregnant women having an *A*/*B* or *B*/*B* genotype. This correlation indicates that *B* haplotypes protect against pre‐eclampsia. For pregnancy of *A/A* mothers, the risk increases further if the fetus has C2**^+^**HLA‐C, especially if the mother lacks C2**^+^**HLA‐C. This correlation strongly implies that interaction of fetal C2**^+^**HLA‐C with KIR2DL1 is the cause of the increased risk 9. Study of European cohorts has shown that the protective effect of a maternal *B* haplotype is mediated by KIR2DS1, the activating C2 receptor 10. Thus the interaction between fetal C2 and activating KIR2DS1 can counter that of fetal C2 with inhibitory KIR2DL1, and in this way reduce the likelihood of pre‐eclampsia 12.

Similar study of a Ugandan cohort of pregnant women correlated a different activating receptor, KIR2DS5, with protection from pre‐eclampsia. Of 11 *KIR2DS5* alleles present in this cohort of sub‐Saharan Africans, KIR2DS5*006 was associated with significant protection 11. That KIR2DS5*006 appears to counter the interaction between C2 and KIR2DL1, implies that KIR2DS5*006 is an activating receptor that recognizes the C2 epitope of HLA‐C.

Whereas KIR2DS5 is highly polymorphic in Africans 13 and African–Americans 14 that is not the case for Europeans and other populations outside Africa. In these populations the KIR2DS5*002 allotype dominates 15. Also present in the cohort of Ugandan women studied by Nakimuli et al. 11, the KIR2DS5*002 allotype provided no protection against pre‐eclampsia, consistent with previous cellular and molecular analyses that failed to detect any functional or molecular interaction of KIR2DS5*002 with C1**^+^**HLA‐C, C2**^+^**HLA‐C, or any HLA‐A or HLA‐B variant 8, 16. With this background, the aim of our study was to test the hypothesis that KIR2DS5*006 differs from KIR2DS5*002, and is an activating receptor for the C2 epitope of HLA‐C.

## Results and Discussion

### KIR2DS5*006 is a C2‐specific receptor

**Figure 1:**
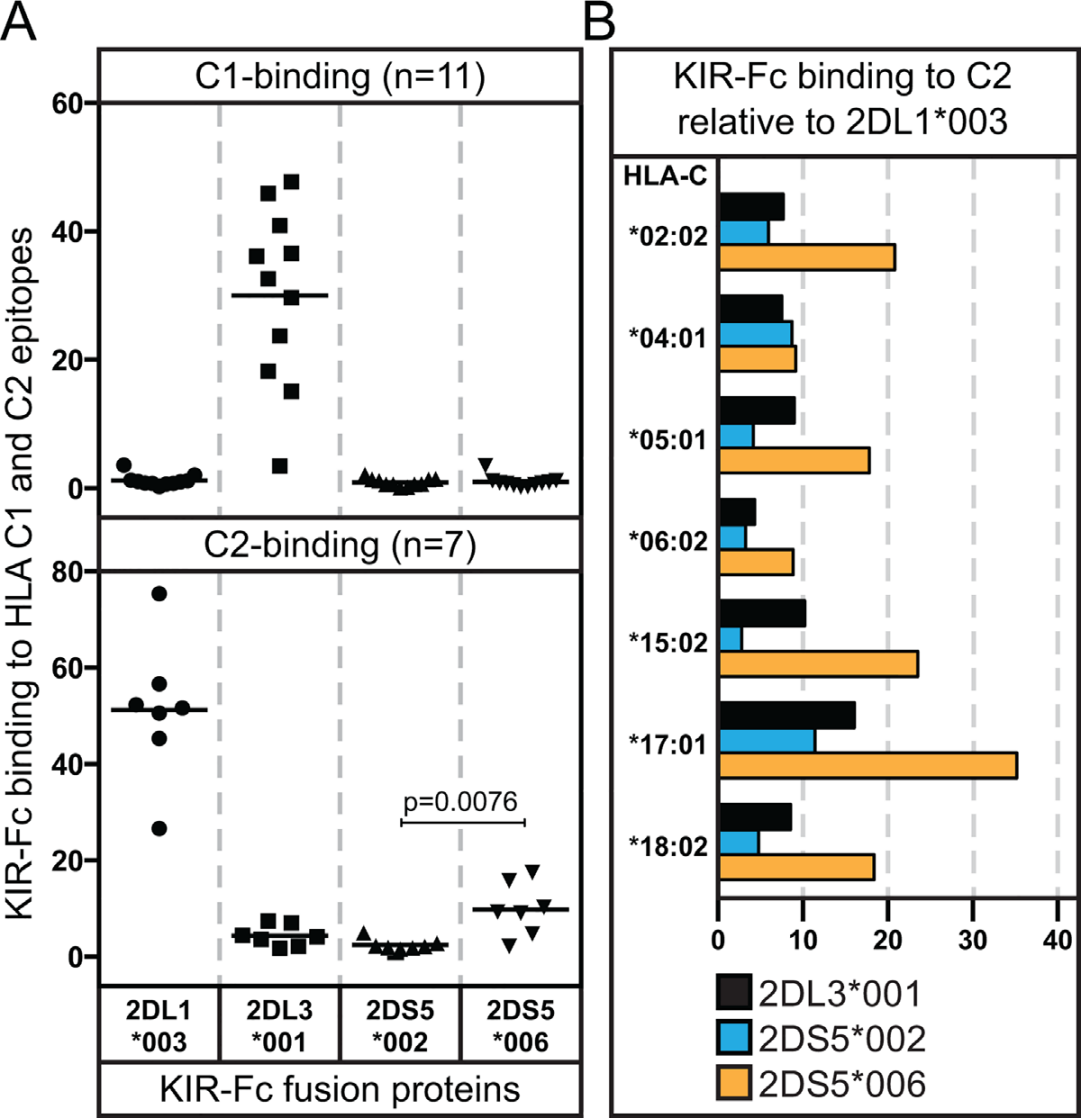
KIR2DS5*006 specifically recognizes the C2 epitope of HLA‐C. (A) Comparison of the binding of KIR2DL1*003, 2DL3*001, 2DS5*002, and 2DS5*006 KIR‐Fc fusion proteins to nine C1**^+^**HLA‐C allotypes and two C1**^+^**HLA‐B allotypes (upper panel) and seven C2**^+^**HLA‐C allotypes (lower panel). Each data point represents the binding to a different HLA class I allotype. For each HLA‐coated bead, the binding data obtained with KIR‐Fc were normalized to that of the W6/32 antibody, which reacts equivalently with all HLA‐A, ‐B and ‐C allotypes (calculation detailed in *Materials and Methods* section). Four independent binding assays were performed for each KIR‐Fc and a representative experiment is shown. The mean binding value is indicated by the horizontal bar. The binding of KIR2DS5*006 to C2^+^HLA‐C is significantly higher than that of KIR2DS5*002 (*two‐tailed paired Student's t‐test, p* *= 0.0076*). The statistical analysis used mean binding values from four independent assays (see Figure S1). (B) Binding of the KIR2DL3*001, 2DS5*002 and 2DS5*006‐Fc fusion proteins to each of the seven C2**^+^**HLA‐C allotypes is shown normalized to that of KIR2DL1*003. Values shown are representative of four independent binding experiments for each KIR‐Fc.

Fc‐fusion proteins corresponding to the D1 and D2 domains of KIR2DS5*002 and KIR2DS5*006 were tested for binding to a panel of 97 Luminex beads 17, each coated with a different HLA‐A, ‐B, or ‐C allotype (Fig. 1). Serving as controls were Fc‐fusion proteins corresponding to C2‐specific KIR2DL1*003 and C1‐specific KIR2DL3*001. None of the KIR‐Fc bound to any HLA‐A allotype. The only observed binding to HLA‐B was of KIR2DL3*001‐Fc with HLA‐B*46:01 and ‐B*73:01, allotypes that have the C1 epitope 5, 18. As expected, KIR2DL3*001 bound strongly to C1**^+^**HLA‐C but weakly to C2**^+^**HLA‐C, and KIR2DL1*003 bound strongly to C2**^+^**HLA‐C but weakly to C1**^+^**HLA‐C.

KIR2DS5*002‐Fc gave no significant binding to either C1**^+^**HLA‐C or C2**^+^**HLA‐C. In contrast, KIR2DS5*006 exhibited little interaction with C1**^+^**HLA‐C, but bound C2**^+^**HLA‐C to a higher level that was statistically significant (Fig. 1A). The mean binding of KIR2DS5*006 to the seven C2**^+^**HLA‐C allotypes was 19% that of C2‐specific KIR2DL1*003. These results demonstrate that KIR2DS5*006 is a C2‐specific receptor, with an avidity around one fifth that of KIR2DL1*003. In contrast, KIRDS5*002 has no significant interaction with C2**^+^**HLA‐C, consistent with previous analyses 8, 16.

KIR2DS5*006 bound the seven C2**^+^**HLA‐C allotypes with variable avidity. For six of the C2**^+^**HLA‐C allotypes, the binding to KIR2DS5*006 exceeds the binding to KIR2DS5*002 or KIR2DL3*001 (Fig. 1B). The exception is HLA‐C*04:01, a common and widespread C2**^+^**HLA‐C allotype, that bound KIR2DL3*001, KIR2DS5*002 and KIR2DS5*006 to a similarly low extent. The strongest binding to KIR2DS5*006 was with HLA‐C*17:01, a common allotype of sub‐Saharan African populations (Fig. 1B), as is C2**^+^**HLA‐C*18:02. The range of KIR2DS5*006 binding to C2**^+^**HLA‐C allotypes is 9–35% that of KIR2DL1*003. This compares to 30–65% for KIR2DS1*002, the most common KIR2DS1 allotype and a well‐characterized C2‐specific activating KIR 4, 6, 19, 20, 21. KIR2DS5*006 is thus seen to be a weaker activating C2 receptor than KIR2DS1*002. In part, the lower avidity of KIR2DS5*006 is likely to be caused by threonine at position 44, because mutation of lysine 44 to threonine in KIR2DL3*001 reduced its avidity by ∼50%, as well as changing its specificity from C1 to C2 22.

### KIR2DS5 polymorphism modulates receptor avidity for C2**^+^**HLA‐C

**Figure 2:**
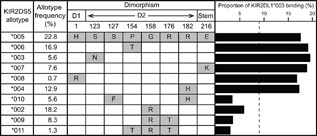
KIR2DS5 allotypes differ in their capacity to recognize C2^+^HLA‐C. Shown are the relative frequencies of the ten KIR2DS5 allotypes observed in the cohort of pre‐eclampsia patients studied by Nakimuli et al. 11, [Frequencies are calculated from *KIR2DS5^+^* individuals only and are not the allele frequencies in the patient population, many of whom lack *KIR2DS5*]. Listed for each allotype are the amino‐acid substitutions that distinguish their extracellular domains (D1 and D2 and Stem) and the binding of their corresponding KIR‐Fc to seven C2^+^HLA‐C allotypes. KIR2DS5*005, the most frequent allotype in this cohort, is set as the consensus sequence with blank boxes indicating sequence identity to the consensus. Shown on the right is the mean binding of each KIR2DS5‐Fc to each C2^+^HLA‐C, normalized to that of 2DL1*003. The dashed vertical line shows KIR2DL3*001‐Fc binding to the same allotypes.

**Figure 3:**
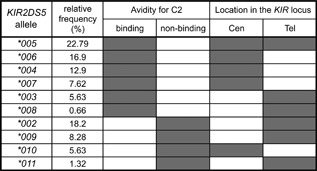
The majority of African KIR2DS5 allotypes recognize C2^+^HLA‐C. Shown are the relative frequencies of the KIR2DS5 allotypes that do, or do not, bind C2^+^HLA‐C. The frequencies are from the cohort studied by Nakimuli et al. 11 and represent relative frequencies of KIR2DS5 allotypes in KIR2DS5^+^ individuals in this population. The *KIR2DS5* alleles distribute between locations in centromeric and telomeric regions of the *KIR* locus. Shown is the distribution between the centromeric and telomeric regions of the *KIR* locus of the alleles encoding KIR2DS5 allotypes that, do or do not, bind C2^+^HLA‐C. KIR2DS5*005 has the highest frequency and is the only allele found in both genomic regions.

Ten of the eleven KIR2DS5 allotypes present in the Ugandan cohort studied by Nakimuli et al. 11 are distinguished by substitutions in the extracellular part of the receptor. Of the eight dimorphisms that distinguish the 10 allotypes, six are in the D2 domain, one is in the D1 domain and one is in the stem (Fig. 2). KIR‐Fc corresponding to the ten KIR2DS5 allotypes were made and compared for binding to HLA‐A, ‐B, and ‐C. Positive reactions were observed only for KIR2DS5‐Fc binding to C2**^+^**HLA‐C. The 10 KIR2DS5 allotypes divide into two groups: one group of six allotypes (2DS5*003, *004, *005, *006, *007, and *008), that bound C2**^+^**HLA‐C to a level that is 15–20% of the KIR2DL1*003 binding and a second group of four allotypes (2DS5*002, *009, *010, and *011) that bound C2**^+^**HLA‐C to an extent that was less than 8% of the KIR2DL1*003 binding (Fig. 2). In the Ugandan cohort we see that 66.5% of the KIR2DS5 allotypes recognize C2**^+^**HLA‐C and 33.5% of them do not (Fig. 3). Thus in Africans a majority of the KIR2DS5 allotypes are active C2**^+^**HLA‐C receptors, whereas in Europeans almost none of them are.

We repeated the analysis described by Nakimuli et al. 11, but with the KIR2DS5 allotypes clustered according to their binding avidities (Table 1). The C2‐binding KIR2DS5 allotypes are seen to protect against pre‐eclampsia compared to the non‐binding allotypes (*p* = 0.01801, OR 0.68). Thus, our functional analysis helps resolve which particular *KIR* gene in the African‐specific *cB01* region protects Ugandan women against pre‐eclampsia.

**Table 1:** Risk of pre‐eclampsia associated with the presence of KIR2DS5 alleles capable of binding to C2^+^HLA‐C (*003,*004,*005,*006,*007,*008) from non‐binding alleles (*002, *009, *010, *011)

*KIR2DS5* alleles	Pre‐eclampsia cases (*N* = 251) *n* (%)	Controls (*N* = 483) *n* (%)	*p*‐value^a^	OR (CI)
*003,*004,*005,*006,*007,*008	82 (32.7)	201 (41.6)	0.01801	0.68 (0.49–0.94)
*002, *009, *010, *011	44 (17.5)	101 (20.9)	NS	0.80 (0.54–1.19)

^a^ Fisher's exact test with mid‐p adjustment.

As a group the six KIR2DS5* allotypes that bind C2 are associated with protection from pre‐eclampsia, but in testing the allotypes individually only KIR2DS5*006 is significantly protective 11. A key question is why the other C2‐binding allotypes (2DS5*003, *004, *005, *007, and *008) are not associated with protection. One possibility is that there are differences in the expression of KIR2DS5 allotypes, which change the amount of receptor at the NK cell surface or the frequency of uterine NK cells expressing the receptor. A second possibility is that other KIR2DS5 allotypes do provide protection but because of their low frequencies their effects did not reach statistical significance in the study of Nakimuli et al. 11. Larger cohorts will address this question as well as the contributions to pre‐eclampsia of different C2**^+^**HLA‐C ligands in the fetus.

KIR2DS5*005 has the consensus sequence of all *KIR2DS5* alleles. It is also the only allele found at both the centromeric and telomeric locations of the *KIR2DS5* gene within the *KIR* locus. These properties point strongly to KIR2DS5*005 being the common ancestor of all other *KIR2DS5* alleles. That KIR2DS5*005 is a high‐binding allotype implies that KIR2DS5*002 and the other low‐binding receptors are derived forms, having acquired substitutions that reduced their avidity for C2**^+^**HLA‐C. Phenylalanine 127 and arginine 158 correlate with conversion from high to low binding. Comparison of KIR2DS5*002 and KIR2DS5*009 shows how threonine 176 augments the reduction caused by arginine 158 (Fig. 2). In addition to this affect on C2 binding, Steiner et al. 23 showed that arginine 158, the only difference between KIR2DS5*002 and KIR2DS5*005, causes intracellular retention of KIR2DS5*002, as well as KIR2DS5*009. They attribute this effect to arginine 158 interfering with glycosylation of asparagine 157 23. Despite these deleterious properties, cells expressing KIR2DS5*002 can transmit activating signals when incubated with an antibody that binds KIR2DS5 16.

Comparison of the organization of the *KIR* locus in humans and other hominoids shows that lineage III *KIR* genes originated in the centromeric region. During human evolution, about 1.7 million years ago, a duplication of the centromeric progenitor of the *KIR2DS5* and *KIR2DS3* genes introduced a copy of the *KIR2DS3/5* progenitor into the telomeric region 24. Subsequent divergence resulted in the distinctive *KIR2DS3* and *KIR2DS5* allelic lineages that have an average of 18 amino acid differences compared to an average of two differences between pairs of sequences in the same lineage. The substitutions that distinguish *KIR2DS3* and *KIR2DS5* are spread throughout the sequence, suggesting that the two *KIR2DS3/5* genes diverged independently. Having the consensus sequence points to KIR2DS5*005 as the ancestor of all extant *KIR2DS5* alleles. The location of KIR2DS5*005 in both the centromeric and telomeric intervals could be interpreted as evidence for KIR2DS5*005 being the ancestor of all *KIR2DS3* and *KIR2DS5*. However, the large number of differences distinguishing these allelic lineages indicates that the dual position of KIR2DS5*005 is more likely the product of a subsequent unequal recombination event that occurred after the divergence of the *KIR2DS3* and *KIR2DS5* allelic lineages. Of the five KIR2DS5 allotypes encoded in the centromeric region, four recognize C2**^+^**HLA‐C, whereas only three of the six KIR2DS5 allotypes encoded in the telomeric region recognize C2**^+^**HLA‐C (Fig. 3). Thus there has been increased attenuation of telomeric *KIR2DS5* alleles than centromeric *KIR2DS5* alleles. Of the eight substitutions that distinguish other KIR2DS5 allotypes from KIR2DS5*005 (the ancestral allele), three (F127, R158, and T176) caused major loss of ligand binding, two had little effect (R1 and H182) and three gave modest increases in ligand binding (N123, T154, and K216). In their comparison of eight KIR2DS5 allotypes, Steiner et al. 25 showed that KIR2DS5*002 and KIR2DS5*009 have low cell‐surface expression, because they are largely retained inside cells, whereas KIR2D5*003, *004, *005, *006, *007, and *008 have higher cell‐surface expression. This difference correlates with the presence of arginine 158 in KIR2DS5*002 and KIR2DS5*009 (Fig. 2). Acquisition of this residue has had two critical effects, reducing cell‐surface expression and reducing avidity for C2. It has been proposed that arginine 158 exerts these effects by interfering with the glycosylation at asparagine 157. Asparagine 123, which distinguishes KIR2DS5*003 from KIR2DS5*005, creates a glycosylation site and increases the level of cell‐surface expression of KIR2DS5*003. Thus the increased expression could be due to the substitution of serine for asparagine at position 123, to the carbohydrate attached to asparagine 123 or combination of these two factors. Cell‐surface expression of KIR2DS5*002 is also increased by having asparagine at position 123 instead of serine 25.

### KIR2DS5*002 is in strong linkage disequilibrium with KIR2DS1*002

In non‐African populations, KIR2DS5*002 is part of a conserved, telomeric *KIR* haplotype that consists of KIR2DL4*00501, 3DS1*013, 2DL5A*001, 2DS5*002, 2DS1*002, and 3DL2*00701. This conservation is well illustrated by the IHWG panel of *HLA* homozygous cells lines that are predominantly of European origin. Although homozygous for *HLA*, these cells are almost all heterozygous for the *KIR* locus. Their *KIR* haplotypes have been determined at allele‐level resolution by complete nucleotide sequencing 26. Among the 194 *KIR* haplotypes of the 97 cell lines, are 31 that contain KIR2DS5*002. Of these, 28 have the conserved telomeric haplotype. The other three haplotypes differ only from the conserved haplotype by their *KIR3DL2* allele. One additional telomeric haplotype contains a *KIR2DS5* gene. This haplotype is the same as the conserved haplotype with the exception of the *KIR2DS5* allele which is KIR2DS5*015. This allele differs by a single substitution that results in substitution of aspartate by asparagine at position 271. Thus all haplotypes with KIR2DS5*002 have KIR2DS1*002 as the neighboring gene, which encodes an activating C2 receptor that is stronger than any of the KIR2DS5 allotypes. This raises the possibility that emergence of KIR2DS1 directly led to the attenuation of KIR2DS5, as is most clearly manifest for KIR2D5*002.

In a variant of the conserved telomeric haplotype, KIR2D5*002 is replaced by KIR2DS3*002 and KIR2DL5A*001 is replaced by KIR2DL5A*005. *KIR2DS3* has sequence similarity with *KIR2DS5* and also encodes KIR with threonine 44. There is considerable evidence for the attenuation of KIR2DS3 27 which is poorly expressed at the cell surface. This too could have been caused by emergence of KIR2DS1. The third human *KIR* that encodes threonine 44 is the inactivated *KIR2DP1* gene, which once encoded two lineages of inhibitory KIR allotypes, one having lysine 44 and specificity for the C1 epitope, the other having threonine 44 and specificity for the C2 epitope 28. The complete or partial demise of the human KIR with threonine 44, could have been driven by the emergence of the stronger KIR2DL1 and KIR2DS1 C2‐specific receptors with methionine 44. KIR with threonine 44 have been found only in the human species and may represent an evolutionary intermediate that allowed the human KIR system to recover from the loss of KIR diversity that accompanied human speciation 28.

The rarity of the conserved telomeric KIR2DS5*002 haplotype in anthropologically well characterized African populations (Nemat‐Gorgani et al., unpublished data and 13) and its prevalence in European populations, raises the possibility that modern Europeans acquired this haplotype from archaic Europeans, such as Neandertals, a phenomenon that we previously considered in the context of the KIR3DS1*013 component of the haplotype 29. A major benefit of the haplotype was likely conferred by KIR2DS1 in reducing the incidence of pre‐eclampsia and related pregnancy syndromes 9. On the other hand, study of European pregnancies has shown that excessive activation caused by paternal C2^+^HLA‐C interacting with maternal KIR2DS1 can lead to babies with high birth‐weight and the potential complication of obstructed labor, which in the absence of surgical intervention can cause death of both mother and child 30. Because KIR2DS5*006 has a lower avidity for C2 than KIR2DS1, this activating C2 receptor of Africans is predicted to have a reduced propensity for excessive activation than KIR2DS1 in Europeans. Indeed, as has recently been reviewed 12, Africans have fewer high birth‐weight babies than Europeans with the result that pre‐eclampsia and other conditions associated with low birth weight, are more prevalent than obstructed labor.

## Material and Methods

### Ethics statement

The experiments reported here involved no animal or human subjects. Results presented in Table 1 regarding the Ugandan cohort are a reanalysis of previously published data 11.

### Sequences of KIR2DS5 alleles and proteins

The amino acid sequences of 12 KIR2DS5 allotypes are deposited in the Immunoreceptor Polymorphism Database (IPD) 31. These sequences were first aligned automatically in Seaview 4.4.0 32 and at positions where the alignment could not be resolved it was manually aligned within the same program. KIR‐Fc fusion proteins, corresponding to 10 of the KIR2DS5 allotypes, were made. One KIR‐Fc represented both KIR2DS5*006 and KIR2DS5*012, because they have identical D1 and D2 domains, differing only by a single amino acid substitution in the signal sequence. The KIR2DS5*001 sequence was not used to make a KIR‐Fc because it is unlikely to represent a natural KIR2DS5 allotype, does not fold properly and is retained inside cells 23, 25. Although it was the first *KIR2DS5* sequence determined 33 subsequent analyses failed to identify any individual or cell line that types for the KIR2DS5*001 allele 34, 35. That no confirmation of KIR2DS5*001 has occurred in the 20 years since it was first reported 33, argues strongly for the four coding nucleotide substitutions (in codons −20, 111, 164, and 174) that distinguish the coding region of KIR2DS5*001 from those of all other *KIR2DS5* alleles, being artifacts of sequencing error. It is time for KIR2DS5*001 to be retired, in the same way that HLA‐A*24:01 and HLA‐B*07:01 were retired 36.

### KIR‐Fc fusion proteins and assay of their binding to HLA class I

A sequence encoding residues 1–224 of the mature KIR2DS5*005 protein was synthesized by Genscript (Piscataway, NJ). This KIR2DS5*005 construct was used as the template for mutagenesis, from which we made equivalent constructs corresponding to nine other *KIR2DS5* alleles. Site‐directed mutagenesis of KIR2DS5*005 used the QuickChange kit (Stratagene, La Jolla, CA), according to the manufacturer's instructions. The *KIR2DS5* constructs were cloned into the baculovirus transfection vector, pVL1393 (Expression Systems, Davis, CA). The sequences of these constructs were determined and verified to be correct. Together with linearized baculovirus DNA (Expression Systems) the constructs were transfected into Sf9 insect cells to generate baculoviruses. The baculoviruses were used to transduce Hi5 insect cells and express KIR‐Fc fusion proteins, as is fully detailed in our methods paper 17. The integrity of KIR‐Fc fusion protein folding was assessed by flow cytometry as described 17. The Sf9 and Hi5 cells were kindly provided by Chris Garcia, Stanford University.

KIR‐Fc fusion proteins corresponding to KIR2DS5 allotypes and site‐directed mutants, as well as the KIR2DL1*003 and KIR2DL3*001 controls, were tested for binding to a panel of 97 microbeads, each coated with one of 31 HLA‐A, 50 HLA‐B, and 16 HLA‐C allotypes (LabScreen Single‐Antigen beads lot #8, One Lambda, Kittridge, CA). To account for differences in the amount of HLA class I protein coating each of the 97 beads, the binding of KIR‐Fc fusion proteins was normalized to that of W6/32, a monoclonal antibody detecting an epitope shared by all HLA class I. Normalized values were calculated using the formula: (specific binding‐bead background)/(W6/32 binding‐bead background). As a comparison for KIR2DS5 binding, we used our published binding data for KIR2DS1*002, the known activating receptor that is specific for C2 4, 8.

### Genetic analysis

Samples of DNA from a case‐control study of pre‐eclampsia involved 738 pregnant women at Mulago Hospital, Kampala in Uganda were typed for *KIR* and *HLA‐C* variants including for presence/absence of *KIR2DS5* alleles 11, 37. Categorical data was analyzed with chi‐square and Fisher's exact test with two‐tailed mid‐p adjustment. A *p*‐value of ≤0.05 was considered to be statistically significant. The magnitude of the effect was estimated by conditional maximum likelihood estimate of Odds Ratio (OR) for the mid‐p exact test and their 95% confidence intervals (CI).

## Conflicts of Interest

The authors declare no commercial or financial conflict of interest.

## Supporting information

Additional supporting information may be found in the online version of this article at the publisher's web‐site

**Figure S1**. Binding of KIR2DS5‐Fc to HLA‐C allotypes is specific to C2 epitopes. (A‐B) Individual KIR2DS5‐Fc were incubated with a panel of 97 microbeads, each coated with one of 31 HLA‐A, 50 HLA‐B and 16 HLA‐C allotypes. KIR2DS5‐Fc binding normalized to W6/32 binding (described in Materials and Methods) is shown in (A) C2^+^ HLA‐C and (B) C1^+^ HLA‐C and C1^+^ HLA‐B. Data is displayed as mean ± SD and comes from a minimum of two independent binding assays for each KIR2DS5‐Fc. No binding was observed to any HLA‐A or the remaining 48 C1^‐^ HLA‐B allotypes (data not shown).
