# Supplementary figures and images for: KIR2DS5 allotypes that recognize the C2 epitope of HLA‐C are common among Africans and absent from Europeans
Source: Immun Inflamm Dis. 2017 Jul 6;5(4):461–8. doi: 10.1002/iid3.178 (PMC5691316; doi:10.1002/iid3.178)

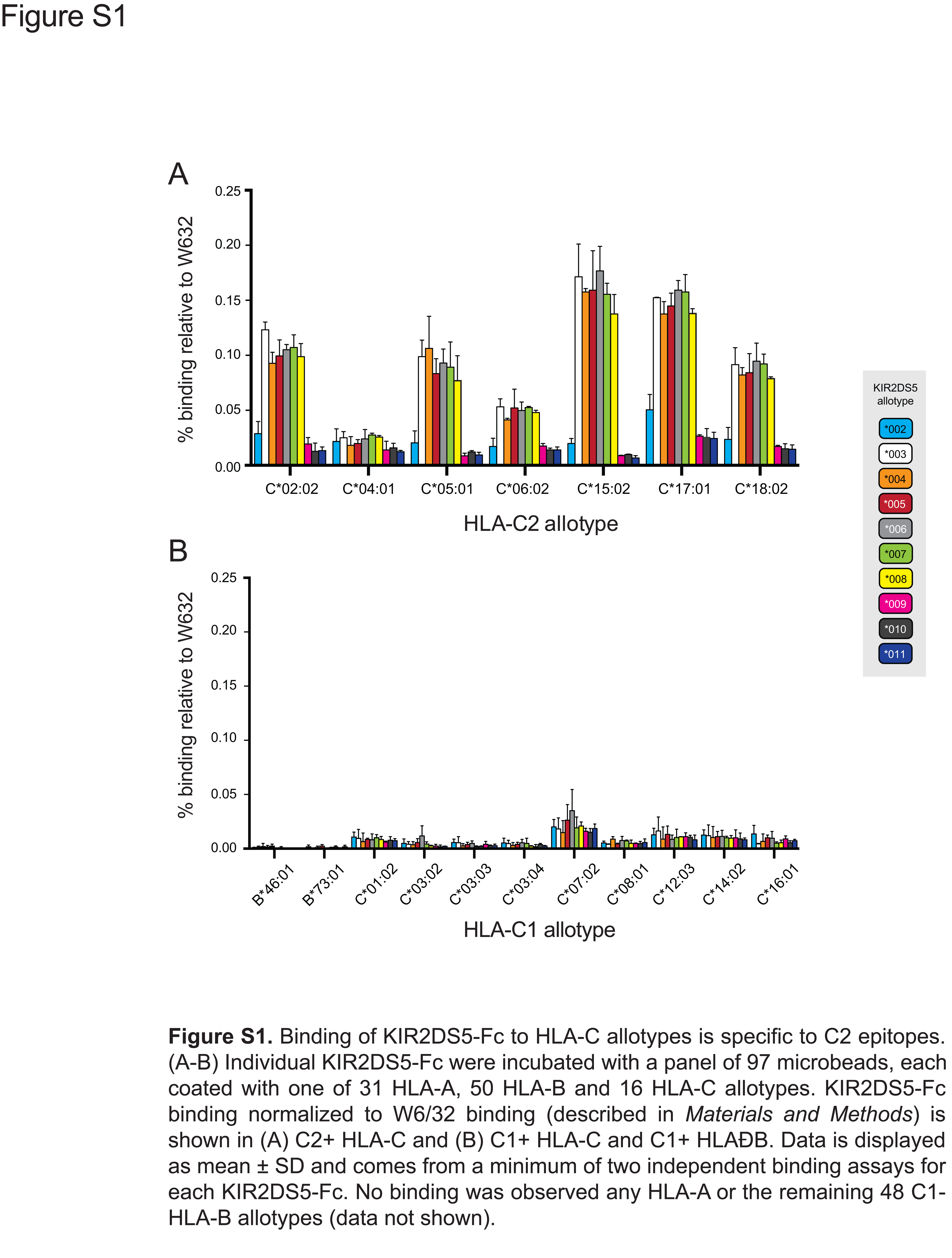

Supplement: Supplementary file 1 — Figure S1. Binding of KIR2DS5‐Fc to HLA‐C allotypes is specific to C2 epitopes. (A‐B) Individual KIR2DS5‐Fc were incubated with a panel of 97 microbeads, each coated with one of 31 HLA‐A, 50 HLA‐B and 16 HLA‐C allotypes. KIR2DS5‐Fc binding normalized to W6/32 binding (described in Materials and Methods) is shown in (A) C2+ HLA‐C and (B) C1+ HLA‐C and C1+ HLA‐B. Data is displayed as mean ± SD and comes from a minimum of two independent binding assays for each KIR2DS5‐Fc. No binding was observed to any HLA‐A or the remaining 48 C1‐ HLA‐B allotypes (data not shown). [file IID3-5-461-s001.tif]
